# Smoking, disease characteristics and serum cytokine levels in patients with primary Sjögren’s syndrome

**DOI:** 10.1007/s00296-018-4063-8

**Published:** 2018-05-30

**Authors:** Peter Olsson, Kristin Skogstrand, Anna Nilsson, Carl Turesson, Lennart T. H. Jacobsson, Elke Theander, Gunnar Houen, Thomas Mandl

**Affiliations:** 1 Department of Clinical Sciences, Malmö, Rheumatology, Lund University, Malmö, Sweden; 2 Department of Rheumatology, Skåne University Hospital, Malmö, Sweden; 3 Department of Congenital Disorders, Center for Neonatal Screening, Statens Serum Institut, Copenhagen, Denmark; 4 Department of Rheumatology, Linköping University Hospital, Linköping, Sweden; 5 Department of Rheumatology and Inflammation Research, The Sahlgrenska Academy, University of Gothenburg, Göteborg, Sweden; 6 Janssen Cilag, Solna, Sweden; 7 Department of Autoimmunology and Biomarkers, Statens Serum Institut, Copenhagen, Denmark; 8 Reumatologmottagningen SUS Malmö, Jan Waldenströms gata 1B, 20502 Malmö, Sweden

**Keywords:** Sjögren’s syndrome, Cytokines, Cigarette smoking, Autoimmune diseases

## Abstract

Smoking affects several disease processes. Epidemiological studies have previously found a negative association between primary Sjögren’s syndrome (pSS) and smoking. The aim of this study was to examine whether markers of disease activity and cytokine expression in pSS patients differ between ever and never smokers. Fifty-one consecutive pSS patients and 33 population controls were included in the study. Clinical and standard laboratory parameters were registered. Serum cytokines (IL-1β, IL-2, IL-3, IL-4, IL-6, IL-8, IL-10, IL-12, IL-17, IL-18, IL-33, IFN-α, IFN-γ, TNF-α, EGF, BAFF, Fas-ligand, RANTES, TGF-β1) were assessed. A positive lip biopsy was less prevalent among ever smoking patients compared to never smokers (81 vs 100%; *p* = 0.03). However, except for TNF-α, which was higher in ever smokers, no differences in cytokine levels were found when comparing ever and never smoking pSS patients. Furthermore, no significant differences were found between ever and never smoking patients in the ESSDAI total score, IgG levels, or complement levels. However, IL-6, IL-12, IL-17 and IL-18 were significantly increased in pSS patients compared to controls. In this study, a negative association between ever smoking and positive lip biopsy was found, confirming previous reports. Expected differences in cytokine levels compared to controls were noted, but no major differences were found between ever and never smoking pSS patients. Taking into account the negative association between pSS diagnosis and smoking in epidemiological studies, possible explanations include a local effect of smoking on salivary glands rather than systemic effects by cigarette smoke.

**Electronic supplementary material:**

The online version of this article (10.1007/s00296-018-4063-8) contains supplementary material, which is available to authorized users.

## Introduction

Primary Sjögren’s syndrome (pSS) is an autoimmune disease characterised by inflammation and destruction of the exocrine glands, classically causing oral and ocular dryness. Extraglandular organs such as lungs, kidneys, skin and nervous system may also be involved in the disease [1, 2]. The underlying pathogenesis is poorly understood, but one hypothesis states that viral triggering in combination with genetic susceptibility may result in an autoimmune attack directed against endogenous proteins and apoptotic material. The reaction results in an upregulation of proinflammatory cytokines, such as IFNα, IFNγ, and BAFF, as well as in an activation of B- and T-cells with a subsequent destruction of exocrine glands [3].

Smoking has well known negative effects, e.g. increased risk of several malignancies, development of COPD, cardiovascular disease, and rheumatoid arthritis, but on the other hand ameliorates clinical symptoms in other diseases, e.g. Behcet’s disease, ulcerative colitis and Parkinson’s disease [4–11]. An immunosuppressive effect by the cigarette smoke has been suggested, since not only smoke but also nicotine alone has been shown to exert effects on the immune system [12, 13].

A negative association between pSS diagnosis and smoking has previously been reported by our group and others [14–17]. It is not known whether these observations represent causality. A negative association between smoking and presence of anti-SSA antibodies and presence of focal sialadenitis in lower lip biopsies, respectively, have also been demonstrated which supports the possibility that smoking affects the disease itself [14, 16]. For example, one can argue that smoking could mask the disease by reducing the foci formation whereby patients might not fulfil the AECG or ACR/EULAR criteria, or that smoking may inhibit the development of the disease. Previous studies have shown a dose dependent effect of smoking on the presence of focal sialadenitis and have also shown that although there are no foci formations in biopsies from smoking pSS patients, there are minor inflammatory infiltrates including CD20+ cells that are not present in healthy controls, thus suggesting that smoking might inhibit the migration of lymphocytes into the salivary glands [14, 16, 18]. Cytokine aberrations have previously been demonstrated in pSS patients compared to healthy controls [19], especially in pSS patients in whom germinal centre formation has been demonstrated in salivary gland biopsies [20]. As reviewed by Arnson et al., cigarette smoke may also affect both immune cells and cytokine production [12]. To our knowledge, no other studies have been performed analysing cytokine expression in relation to smoking in pSS patients.

Taken together, the relation between pSS and smoking should be further explored. The aims of this study were (1) to investigate differences in markers of disease activity and serum cytokine levels between ever and never smoking pSS patients and (2) to assess differences in cytokine levels between pSS patients vs population-based controls.

## Materials and methods

### Patients and controls

At the Department of Rheumatology, Skåne University Hospital Malmö, Sweden, consecutive patients with pSS have been followed and registered since 1984. The register entailed 380 patients at the time of the study. Fifty-one consecutive outpatients who had been diagnosed with pSS by a specialist in rheumatology and fulfilled the American-European Consensus Group (AECG) criteria [21], seen from May to December in 2012, at our department were included in the study (females 49/51, median age 61, IQR: 52; 69). Clinical and laboratory parameters were assessed according to a structured protocol including ESSDAI (EULAR Sjögren’s Syndrome Disease Activity Index) and ESSPRI (EULAR Sjögren’s Syndrome Patient Reported Index) [22]. Median ESSDAI-total value was 7, (IQR: 1; 10). Forty of the patients had performed a lower lip biopsy of which 37 were positive. A positive lip biopsy was defined according to the AECG criteria (i.e. ≥ 1 focus of 50 cells or more per 4 mm^2^). Anti-SSA positivity was found in 40/51 patients. Demographic and clinical data on patients and controls are summarised in Table 1. When investigating the effect of smoking on presence of positive lip biopsy and presence of SSA/SSB antibodies, smoking status at the time of diagnosis was used since the biopsies and analysis of SSA/SSB antibodies were performed at the time of diagnosis. The patients were also investigated for presence of chronic obstructive pulmonary disease (COPD) and interstitial lung disease (ILD) as part of another study, as previously reported [23]. Briefly, COPD was defined according to the Global Initiative for Lung Disease (GOLD) criteria [24] and ILD was defined as presence of ground glass attenuation, traction bronchiectasis, or honeycombing in high-resolution CT scans.

**Table 1:** Demographic characteristics of 51 consecutive patients and 33 controls

	Cases (*n* = 51)	Controls (*n* = 33)
Age, years	61 (52; 69)	47 (39; 61)
Sex, females	49/51 (96)	19/33 (58)
Current/not current smokers	4/51 (8)	7/33 (21)
Ever/never smokers	24/51 (47)	NA
Fulfilling the AECG for pSS	51/51 (100)	NA
Fulfilling the ACR/EULAR criteria for pSS	51/51 (100)	NA
Disease duration, years	12 (6; 18)	NA
Anti-SSA antibody seropositives	40/51 (78)	NA
Anti-SSB antibody seropositives	24/51 (47)	NA
ANA seropositives	40/51 (78)	NA
RF seropositives	26/51 (51)	NA
IgG, g/l	13.0 (10.1; 15.5)	NA
C3, g/l	1.01 (0.86; 1.20)	NA
C4, g/l	0.18 (0.13; 0.21)	NA
Lower lip biopsy, focus score ≥ 1%	37/40 (93)	NA
ESSPRI total score	6 (5; 7)	NA
ESSDAI total score	7 (1; 10)	NA
Nonexocrine symptoms/signs, any of the below %	25/51 (49)	NA
Lymphadenopathy and/or lymphoma ever %	3/51 (6)	NA
Arthritis ever %	4/51 (8)	NA
Cutaneous symptoms ever %	10/51 (20)	NA
Interstitial lung disease ever^a^ %	9/51 (18)	NA
Chronic obstructive lung disease ever %	21/51 (41)	NA
Renal involvement ever %	4/51 (8)	NA
Myositis ever %	0/51 (0)	NA
Peripheral nervous system involvement ever %	1/51 (2)	NA
Raynaud phenomenon ever %	4/51 (8)	NA

Disease characteristics of the 51 consecutive patients with pSS. Values are presented as n/n available (%) or median (IQR) unless otherwise specified

*pSS* primary Sjögren’s syndrome, *AECG* American-European Consensus Group criteria, *ACR* American College of Rheumatology, *EULAR* European League Against Rheumatism, *ANA* antinuclear antibody, *RF* rheumatoid factor, *IgG* immunoglobulin G, *C3* complement factor 3, *C4* complement factor 4, *ESSPRI* EULAR Sjögren Patient Reported Index, *ESSDAI* EULAR Sjögren Disease Activity Index, *EULAR* European League Against Rheumatism, *ILD* interstitial lung disease

^a^Defined as presence of peripheral traction bronchiectasis, honey combing or ground glass opacities

Population-based controls, living in the city of Malmö or its surroundings were randomly selected from the Swedish population register and asked via mail if they were willing to participate in the study. If informed consent was received, the subject was invited to the Department of Rheumatology outpatient clinic, where data on age, sex, medical history, medication, present pregnancy and current smoking were registered, and a physical exam was performed. Thirty-three controls were included (females 19/33, median age 47, IQR 39; 61).

### Cytokine analyses

Serum samples from patients and controls were obtained and stored at − 80 °C until analysis. Since, to our knowledge, no other studies on cytokine expression in relation to smoking and pSS previously have been published, the selection of cytokines was based on findings in previous studies analysing cytokines in pSS patients compared to healthy controls (e.g. BAFF, IFNg, IFNa, EGF) and cytokines stimulating different pathways: T-cell activating cytokines (e.g. RANTES, IL-2), B-cell activating cytokines (e.g. BAFF, IL-4, IL-10, IL-6), the Th-17 pathway (IL-17), and general proinflammatory cytokines (e.g. IL-1B, TNF-α, IL-6, IFNg).

The serum samples were analysed with four different panels, all using the Meso-Scale platform. Panel 4 was purchased from Meso-Scale for Interferon-α2a (IFN-α2a Ultra-sensitive kit, K151ACC). Panel 1–3 were in-house made assays. Panel 1 included B-cell activating factor (BAFF), Epidermal Growth Factor (EGF), Fas-ligand, Interleukin-3 (IL-3), IL-33, Regulated upon Activation, Normal T-cell Expressed and presumably Secreted (RANTES), and Transforming growth factor β 1 (TGF-β1). Panel 2 included IFN-γ, IL-2, -6, -8, -10, -12, -17, -18, -1β, tumour necrosis factor-α (TNF-α). Panel 3 was IL-4. Further details on these assays are given in the Supplementary text. Concentrations were calculated with Discovery Workbench software (Meso-Scale) from calibration curves using four-parameter logistic fit.

Based on previous reports, the panels were assessed for proneness to interaction with heterophilic antibodies using pooled IgM/IgA rheumatoid factor (RF) positive sera and pooled healthy control sera with and without HBR Plus (Scantibodies Laboratory, Santee, CA, USA) without any significant difference in cytokine levels [25, 26]. Subsequent analyses were performed without any additional blocker.

### Statistics

When analysing differences in cytokine levels between ever and never smokers as well as between cases and controls the Mann–Whitney *U* test was used. Spearman’s rank test was used for correlations. Chi^2^ test was used for comparison of binary parameters. A *p* value below 0.05 was considered significant for all analyses. Separate analyses included cases with shorter disease duration (above median) and anti-SSA seropositive patients.

The statistic calculations were performed using SPSS version 22 for Macintosh.

## Results

Amongst the 51 patients, 47% were ever smokers (8% current smokers, 39% former smokers) (Table 1). Amongst ever smokers at the time of diagnosis, significantly fewer patients had a focal sialadenitis (81 vs 100%; *p* = 0.03) (Table 2). The ESSDAI total score, the ESSPRI total score, IgG, C3, and C4 levels did not significantly differ between ever and never smoking pSS patients (Table 2). Levels of IL-6, IL-12, IL-17 and IL-18 were significantly increased in pSS patients compared to controls whilst no major differences between pSS patients and controls for the other cytokines were found (Table 3).

**Table 2:** Comparison of clinical parameters, IgG levels and complement levels between never smoking and ever smoking pSS patients

	Ever smokers (pSS) *n* = 24	Never smokers (pSS) *n* = 27	*p* value^+^
Focal sialadenitis	14/17 (82)	23/23 (100)	0.03*
Anti-SSA- and or SSB-positive	20/24 (83)	20/27 (74)	0.43
ESSDAI	7.5 (1.5; 10)	7 (1; 11)	0.85
ESSPRI	6 (5; 7)	6 (4; 8)	0.68
IgG (g/l)	12.9 (10.1; 17.2)	13.0 (10.1; 15.2)	0.62
C3 (g/l)	1.02 (0.92; 1.22)	0.99 (0.84; 1.16)	0.60
C4 (g/l)	0.19 (0.13; 0.21)	0.16 (0.13; 0.20)	0.40

Values are presented as median (IQR) or n/n available (%)

*p < 0.05

^+^Mann–Whitney *U* test

**Table 3:** Comparison of cytokine levels between pSS patients and controls

	Cases pg/ml, median (IQR) *n* = 51	Controls, pg/ml, median (IQR) *n* = 33	*p* value^+^
IL-1β	0 (0; 0)	0 (0; 0)	0.92
IL-2	0 (0; 25.0)	0 (0; 24.4)	0.86
IL-3	0 (0; 67.8)	0 (0; 88.7)	0.84
IL-4	0 (0; 0)	0 (0; 0)	0.20
IL-6	25.2 (14.0; 30.9)	15.3 (10.6; 22.0)	0.003**
IL-8	19.9 (15.9; 22.8)	16.7 (14.1; 20.8)	0.06
IL-10	0 (0; 0)	0 (0; 0)	0.18
IL-12	7.4 (0; 10.8)	0 (0; 8.3)	0.02*
IL-17	0 (0; 51.2)	0 (0; 0)	0.002**
IL-18	294 (187.7; 500.3)	214.5 (119.0; 297.5)	0.008**
IL-33	11.2 (0; 16.1)	12.5 (0; 17.7)	0.62
IFN-α	0 (0; 0)	0 (0; 0)	0.53
IFN- γ	0 (0; 1.3)	0 (0; 1.0)	0.43
TNF-α	12.1 (5.7; 16.9)	8.5 (5.9; 11.5)	0.14
BAFF	265.6 (182.4; 376.2)	276.1 (142.9; 391.6)	0.79
EGF	139.8 (60.0; 227.1)	136.4 (93.2; 177.3)	0.89
Fas ligand	9.5 (7.2; 15.3)	11.4 (6.9; 15.2)	0.48
RANTES	15673.7 (11374.1; 25397.6)	17073.0 (13345.9; 20037.8)	0.56
TGF-β1	22.4 (6.7; 35.6)	12.7 (9.2; 36.6)	0.75

*p < 0.05

**p < 0.01

^+^Mann–Whitney *U* test

When comparing ever and never smoking pSS patients, only TNF-α levels were significantly higher in the former group (Table 4). Also, when analysing only anti-SSA positives as well as pSS patients with shorter than median disease duration amongst pSS patients, a similar lack of association was found. No significant difference was observed in cytokine levels between patients with or without COPD, or ILD, respectively.

**Table 4:** Comparison of cytokine levels between never smoking and ever smoking pSS patients

	Ever smokers (pSS) *n* = 24 pg/ml, median (IQR)	Never smokers (pSS) *n* = 27 pg/ml, median (IQR)	*p* value^+^
IL-1β	0 (0; 0)	0 (0; 0)	0.30
IL-2	0 (0; 28.4)	0 (0; 19.2)	0.85
IL-3	0 (0; 0)	0 (0; 96.9)	0.36
IL-4	0 (0; 0)	0 (0; 0)	1.0
IL-6	25.0 (14.4; 29.4)	25.2 (13.0; 36.7)	0.94
IL-8	21.8 (15.9; 24.1)	18.3 (15.5; 21.4)	0.18
IL-10	0 (0; 0)	0 (0; 39.3)	0.74
IL-12	9.7 (5.6; 12.9)	7.1 (0; 9.8)	0.20
IL-17	0 (0; 75.7)	39.0 (0; 46.6)	0.86
IL-18	364.3 (250.4; 659.3)	234.5 (166.3; 500.3)	0.06
IL-33	11.2 (0; 19.3)	11.2 (0; 15.6)	0.95
IFN-α	0 (0; 0)	0 (0; 0)	0.51
IFN-γ	0.7 (0; 1.3)	0 (0; 1.0)	0.58
TNF-α	13.8 (7.2; 20.8)	7.5 (4.9; 15.9)	0.03*
BAFF	256.1 (191.9; 375.2)	265.6 (156.7; 388.3)	0.95
EGF	154.8 (55.4; 252.0)	133.9 (70.8; 202.8)	0.53
FAS ligand	10.3 (7.3; 16.6)	9.2 (6.5; 12.6)	0.50
RANTES	17051.1 (11883.2; 27559.6)	15443.0 (9730.2; 19827.9)	0.46
TGF-β1	21.2 (4.2; 26.9)	22.4 (9.3; 55.0)	0.14

*p < 0.05

^+^Mann–Whitney *U* test

Thirty-three controls (median age 47 (range 39–61 years), 19 females) were included, of whom 21% were current smokers (Table 1). Amongst controls, sex and age correlated poorly to cytokine levels. In patients with pSS, disease duration was negatively correlated to IL-10 (*r* = − 0.32, *p* = 0.02), IL-12 (*r* = − 0.34, *p* = 0.02) and TNF-α (*r* = − 0.40, *p* = 0.004) and positively to TGF-β1 (*r* = 0.29, *p* = 0.04). There were no significant correlations between the ESSDAI total score and serum cytokine levels (data not shown). Current smokers entailed only four patients, therefore statistical analyses were not performed on this group separately.

## Discussion

In this study, a negative association between a history of smoking and focal sialadenitis in patients with pSS was found which is in line with previous reports. The negative association between pSS diagnosis and smoking could be due to the dryness of the oral cavity and eyes potentially causing more irritation by the smoke. Cigarette smoking is also reported to cause reduced salivary rates and alteration of the saliva composition [27, 28]. To the best of our knowledge, there are no studies investigating the effect of smoking on salivary gland biopsies in healthy controls. The reported effect on salivary flow does not reach the levels required for diagnosing pSS but might potentially decrease the already diminished salivary flow in pSS patients, thereby explaining the lower frequency of current smokers amongst pSS patients. Still, it does not explain the lower frequency of focal sialadenitis in ever smoking patients. Since the salivary glands are in close proximity to the inhaled smoke, a possible explanation for this finding could be that cigarette smoke interferes with the local immune response either by nicotine binding to nicotine receptors on immune cells or by other compounds in the inhaled smoke acting anti-inflammatory [12, 13, 29, 30].

Apart from the lower frequency of positive lip biopsy, there were no significant differences in other standard clinical and laboratory characteristics between ever and never smokers. Furthermore, there were no major differences in cytokine levels, except for TNF-α, which was higher in ever smokers. The latter finding should be interpreted with caution. The TNF-α levels were generally low, and if smoking was indeed associated with a higher degree of systemic inflammation, one would expect other proinflammatory cytokines to be significantly increased as well in ever smokers. Also, given the numerous statistical analyses, this single statistical significance should not be over-interpreted.

An increase in several proinflammatory cytokines (IL-6, IL-12, IL-17, IL-18) was observed in pSS patients compared to controls. pSS is a disease characterised by an insidious onset and slow progression of exocrinopathy. Since the exocrine inflammation is mainly local, most previous studies have measured cytokine expression in biopsies [31, 32] and saliva [33] or production by peripheral mononuclear cells [34]. However, several other studies have also shown cytokine aberrations in serum including the increase in IL-6, IL-17, and IL-18 in the current study [35–39]. Furthermore, regarding the observed increase in IL-12 in pSS patients, polymorphisms of the IL12A gene have been shown to be associated with pSS in a genome-wide association-study [40].

The observed difference in cytokine expression between pSS patients and controls in our study is thus an expected finding. However, the lack of difference in BAFF levels between patients and controls was unexpected since BAFF is considered a hallmark of pSS in several studies [41, 42] and considered as a potential biomarker [43]. A possible explanation might be that these consecutive patients had a lower disease activity than patients in previous studies or that the in-house made kit was not specific enough. Problems with analysing BAFF due to lack of specificity for BAFF caused by posttranslational glycosylations or alternative spliced forms have previously been reported [44].

The type I interferon system is activated in pSS and is thought to play an important role in the disease development [45, 46]. Type I interferons consists of at least 17 different subtypes, of which there are 13 different subtypes of IFNα. In this study, IFNα2a was investigated. Despite choosing an ultra-sensitive kit, the majority of samples were below the measurable range. This is a common problem and a reason why mRNA from interferon-sensitive genes, the so-called IFN-signature, is often measured rather than IFNα itself. Unfortunately, mRNA was not available in this study. Analysing the IFN-signature in salivary gland cells or monocytes from pSS patients with different smoking exposures would be interesting, since smoking has been shown to suppress the effect of type I IFNs [47].

Most patients included in the study had a longstanding disease (median disease duration 12 years) and disease duration correlated negatively to the proinflammatory cytokines IL-10, IL-12 and TNF-α and positively to the anti-inflammatory cytokine TGF-ß, indicating that the disease develops towards a less inflammatory state over time.

In line with previous reports [14, 16], we found a lower frequency of positive lip biopsy among ever smokers at the time of diagnosis. However, we did not find evidence that ever smoking affects cytokine expression, IgG levels, complement levels or disease activity, measured by the ESSDAI-score, in pSS patients. It is possible that cytokine concentrations and ESSDAI-scores would have differed between ever and never smokers as well if measured at time of diagnosis. Another possible explanation could be that smoking might affect inflammation locally in the salivary glands rather than the systemic inflammation of the disease. Also, the group of currently smoking patients was small in this study, which makes it difficult to draw conclusions about temporary effects of current smoking on cytokine levels. Since current smokers are underrepresented in epidemiological data, it would be of interest in future studies to compare a larger group of currently smoking pSS patients with former and never smoking patients concerning cytokine levels.

The study has some limitations, including the limited sample size of the study and the low number of current smokers amongst the pSS patients. Furthermore, the majority of the patients had long-standing disease, and we cannot exclude that cytokine patterns, and their relation to smoking history, may be different in recently diagnosed patients. The cross-sectional study design is also a limitation. Finally, the control group was not exactly matched on sex and age. Strengths of the study include the use of consecutive patients in standard follow-up, likely representative of the general pSS population and the validation of the assays concerning possible interference by rheumatoid factor.

In conclusion, there was a lower prevalence of positive lip biopsy among pSS patients with a history of ever smoking which is in accordance with previous studies. No differences in serum cytokine levels between ever and never smoking pSS patients was found. Furthermore, we found increased levels of proinflammatory cytokines (IL-6, IL-12, IL-17, IL-18) in pSS patients compared to controls as well as a negative correlation between disease duration and proinflammatory cytokines indicating that the disease develops to a less inflammatory state over time. In pSS, a local effect of smoking on salivary glands rather than systemic effects of cigarette smoke may explain the previously observed negative association between smoking and pSS.

## Electronic supplementary material

Below is the link to the electronic supplementary material.

Supplementary material 1 (DOCX 21 KB)

## Abbreviations

AECG: American-European Consensus Group
BAFF: B-cell activating factor
EGF: Epidermal growth factor
C3: Complement factor 3
C4: Complement factor 4
COPD: Chronic obstructive pulmonary disease
ESSDAI: EULAR Sjögren’s Syndrome Disease Activity Index
ESSPRI: EULAR Sjögren’s Syndrome Patient Reported Index
GOLD: Global Initiative for Lung Disease
IFN-α2a: Interferon-alpha 2a
IFN-γ: Interferon gamma
IL: Interleukin
ILD: Interstitial lung disease
IgG: Immunoglobulin G
pSS: Primary Sjögren’s syndrome
RF: Rheumatoid factor
RANTES: Regulated upon activation, normal T-cell
anti-SSA: Anti-Sjögren’s syndrome A
anti-SSB: Anti-Sjögren’s syndrome B
TGF-β1: Transforming growth factor-beta 1
Th: T-helper cell
TNF-α: Tumour necrosis factor-α
