# Supplementary material for: Smoking, disease characteristics and serum cytokine levels in patients with primary Sjögren’s syndrome
Source: Rheumatol Int. 2018 May 30;38(8):1503–10. doi: 10.1007/s00296-018-4063-8 (PMC6060795; doi:10.1007/s00296-018-4063-8)
Supplement: Supplementary file 1 — Supplementary material 1 (DOCX 21 KB) [file 296_2018_4063_MOESM1_ESM.docx]

Supplemenary text

Additional information on the in-house made cytokine assays.

For the in-house assays (Panel 1,2 and 3), the plates were coated using U-plex development kits (Meso-Scale 10-plex K15235N for panel 2, 7-plex K15232N for panel 1, 2-plex K15227N for panel 3). The different capture antibodies were first biotinylated using EZ-Link Sulfo-NHS-LC-Biotin (ThermoFisher, 21327), then bound to different linkers 1-10, mixed together to the concentrations 10µg/ml per antibody, added to each plate well (50µl/well) for all plates in the respective panels, and incubated for 1 hour. After washing with washing buffer (PBS containing 0,05% Tween 20), the plates were stored at 4°C until use. Detection antibodies were sulfo-tagged using MSD Gold Sulfo-tag NHS-Ester (Meso-Scale, R91AO-2). All antibodies were purchased from RnD systems. Samples were diluted 1:1 in diluent 7 (containing proteins, blockers and preservatives, Meso-Scale, R54BB) and pipetted in duplicates (25µlx2) on the plates together with calibration curves and high and low controls. Calibrators and controls were prepared by recombinant antigens diluted in diluent 7. The plates were sealed and incubated with shaking for 2 hours, washed 3 times, added the corresponding detection antibodies (25µl to each well, conc. 0,1µg/ml), and incubated with shaking for 2 hours. Finally, the plates were washed, added 150µl 2xRead buffer T (Meso-Scale R92TC) per well, and immediately read at the QuickPlex reader.

Supplemental table 1: Difference in cytokine detection signal in pooled healthy control sera and pooled RF positive sera with and without HBR plus

|  | Control pool | Control pool + HBR plus |  |  | RF sera pool | RF sera pool + HBR Plus |  |
| --- | --- | --- | --- | --- | --- | --- | --- |
|  | Signal | Signal | Ratio |  | Signal | Signal | Ratio |
| BAFF | 737 | 810 | 1.10 | **BAFF** | 737 | 817 | 1.18 |
| EGF | 26061 | 24602 | 0.94 | **EGF** | 32823 | 32052 | 0.98 |
| FAS Ligand | 2843 | 2558 | 0.90 | **FAS Ligand** | 3056 | 2690 | 0.88 |
| IL-3 | 788 | 541 | 0.69 | **IL-3** | 586 | 646 | 1.10 |
| Il-33 | 425 | 300 | 0.71 | **Il-33** | 367 | 303 | 0.83 |
| RANTES | 779085 | 754518 | 0.97 | **RANTES** | 774985 | 741468 | 0.96 |
| TGF-β1 | 4085 | 5153 | 1.26 | **TGF-β1** | 2845 | 3604 | 1.27 |
| IFN-γ | 95 | 71 | 0.75 | **IFN-γ** | 81 | 83 | 1.02 |
| IL-10 | 155 | 157 | 1.01 | **IL-10** | 177 | 171 | 0.97 |
| IL-12 | 92 | 89 | 0.97 | **IL-12** | 96 | 105 | 1.09 |
| IL-17 | 94 | 80 | 0.85 | **IL-17** | 117 | 92 | 0.79 |
| IL-18 | 2294 | 4082 | 1.78 | **IL-18** | 4438 | 6809 | 1.53 |
| IL-1β | 94 | 103 | 1.10 | **IL-1β** | 108 | 107 | 0.99 |
| IL-2 | 70 | 79 | 1.13 | **IL-2** | 99 | 100 | 1.01 |
| IL-6 | 116 | 157 | 1.35 | **IL-6** | 149 | 198 | 1.33 |
| IL-8 | 796 | 714 | 0.90 | **IL-8** | 861 | 752 | 0.87 |
| TNF-α | 134 | 143 | 1.07 | **TNF-α** | 248 | 181 | 0.73 |
| IL-4 | 88,8 | 87,3 | 0.98 | **IL-4** | 87,5 | 80,2 | 0.92 |
